# Patient-reported outcomes in a large community-based pain medicine practice: evaluation for use in phenotype modeling

**DOI:** 10.1186/s12911-015-0164-4

**Published:** 2015-05-28

**Authors:** David A. Juckett, Fred N. Davis, Mark Gostine, Philip Reed, Rebecca Risko

**Affiliations:** Biomedical Research Informatics Core, Clinical and Translational Sciences Institute, Michigan State University, West Fee Hall, East Lansing, MI USA; Michigan Pain Consultants, PC, ProCare Systems, Inc., Grand Rapids, MI USA; ProCare Systems, Inc., Grand Rapids, MI USA

**Keywords:** Chronic pain, Patient-reported outcomes, Factor analysis, Pain severity

## Abstract

**Background:**

An academic, community medicine partnership was established to build a phenotype-to-outcome model targeting chronic pain. This model will be used to drive clinical decision support for pain medicine in the community setting. The first step in this effort is an examination of the electronic health records (EHR) from clinics that treat chronic pain. The biopsychosocial components provided by both patients and care providers must be of sufficient scope to populate the spectrum of patient types, treatment modalities, and possible outcomes.

**Methods:**

The patient health records from a large Midwest pain medicine practice (Michigan Pain Consultants, PC) contains physician notes, administrative codes, and patient-reported outcomes (PRO) on over 30,000 patients during the study period spanning 2010 to mid-2014. The PRO consists of a regularly administered Pain Health Assessment (PHA), a biopsychosocial, demographic, and symptomology questionnaire containing 163 items, which is completed approximately every six months with a compliance rate of over 95 %. The biopsychosocial items (74 items with Likert scales of 0–10) were examined by exploratory factor analysis and descriptive statistics to determine the number of independent constructs available for phenotypes and outcomes. Pain outcomes were examined both in the aggregate and the mean of longitudinal changes in each patient.

**Results:**

Exploratory factor analysis of the intake PHA revealed 15 orthogonal factors representing pain levels; physical, social, and emotional functions; the effects of pain on these functions; vitality and health; and measures of outcomes and satisfaction. Seven items were independent of the factors, offering unique information. As an exemplar of outcomes from the follow-up PHAs, patients reported approximately 60 % relief in their pain. When examined in the aggregate, patients showed both a decrease in pain levels and an increase in coping skills with an increased number of visits. When examined individually, 80-85 % of patients presenting with the highest pain levels reported improvement by approximately two points on an 11-point pain scale.

**Conclusions:**

We conclude that the data available in a community practice can be a rich source of biopsychosocial information relevant to the phenotypes of chronic pain. It is anticipated that phenotype linkages to best treatments and outcomes can be constructed from this set of records.

## Background

### Overview

With the high prevalence of chronic pain in America, plus the corresponding personal, social, and economic burdens from pain [1–3], it is critical to have highly effective tools and treatments available for pain medicine practitioners. There is a growing consensus that evidence-based medicine should be the guide for developing these tools and treatments, but this is difficult to achieve due to the challenges in obtaining sufficient data to deduce linkages between treatments and outcomes [4]. For community-based practitioners, evidence-based medicine must be effective in their population and setting. Therefore, many advocate for pragmatic approaches to meet this need [5] instead of the gold standard, randomized controlled trials because they are expensive, complicated to execute [6], narrow in focus, and not generally suitable for comparative effectiveness evaluation in the community setting [7].

Pragmatic approaches to comparative effectiveness must be built on the knowledge of the patients and their environment. Considering that the number of patient visits in the US per year for all conditions is approximately 190 million [8], patient charts represent a vast, untapped resource that could be used to gather this knowledge [9]. Within this resource lies the collective experience of medical practitioners and their patients. We conjecture that extracting the content from this documentation can provide important background for the development of evidence-based personalized treatment in pain medicine and the opportunity for pragmatic comparative effectiveness evaluations among existing treatments.

Extracting the collective experience of medical practitioners requires comprehensive patient charts containing detailed progress notes and patient reported outcomes (PRO). In the field of chronic pain, this must encompass the patient’s complete biopsychosocial phenotype, the constellation of typical treatments, and the range of outcomes. Pain medicine physicians have long grappled with the issue of understanding how pain affects patients and how objective measures can be created for the subjective characteristics of chronic pain. Implementing such measures has clinical, ethical, legal, and social ramifications [10]. This is something the authors (FD, MG) have struggled with throughout 30 years practice, ultimately leading to the development of a patient self-assessment and care management tool (PRISM^TM^). This was designed to give each patient a consistent voice in their medical record, reflecting the complexities and multidimensional nature of pain and its effects on the whole person. Supplementing this is the physician perspective recorded as text in detailed progress notes following an enhanced SOAP note format and rendered in correspondence form. With these components available, an opportunity now exists to utilize this community-based patient data to improve outcomes [11, 12] and empirically justifying the appropriate use of pharmaceutical and procedural interventions. This can be accomplished by converting the data to actionable information through the use of data mining, natural language processing, medical informatics, and decision support tools.

To utilize this data and move toward the goal of personalized therapy in pain management and eventually to the goal of evidence-based pain medicine, Michigan State University (MSU) has partnered with Michigan Pain Consultants PC (MPC) and ProCare Systems, Inc. to share data and expertise. ProCare developed a physician-driven tool to capture comprehensive patient data from community practice utilizing modern electronic techniques consistent with the drive to move medicine into the ‘measured world’. This public-private partnership between MSU, ProCare, and MPC was established to evaluate and utilize this data repository for research and development in the pervasive, costly, and seemingly underfunded domain of medical pain management. In its initial form, this repository will offer the opportunity for pragmatic comparative effectiveness research, but with development it will allow other research, such as clinical trials and development of decision support models.

The immediate goal is to extract content from the MPC reservoir of patient clinical data to identify person-in-pain phenotypes and their associated intervention outcomes. Two early steps toward this goal are to determine if the ProCare repository of MPC data captures a broad spectrum of patient phenotype characteristics, and if suitable outcome measures can be tracked during the course of patient care. Utilizing the concepts of the biopsychosocial model of chronic pain [13] as a guide, several patient characteristics are desirable to allow phenotype construction. These include: physical, emotional, and social status of a patient; the influence of these attributes on pain intensity; the effects of pain on these attributes; and patient outcomes as declared by the patient and physician. Important outcomes are progressive improvements in pain levels, quality-of-life measures, and satisfaction with treatment. Long term, the goal is to marry the phenotype of the patient in pain with the genotype information now mounting [14]. The combination of this information will help guide patterns of treatment likely to result in best outcomes for a particular set of genotype/phenotype expression. This will reduce the more subjective aspects of pain medicine and will be a major step towards personalized therapy.

The content of MPC’s clinical records comprises practice management data, results from detailed patient questionnaires, and detailed progress notes, all derived from approximately 95,000 patient visits per year. Previous work has described the nature of the progress note dictations [15] and early methods for extracting content. Current work is underway to examine the intake (pre-treatment) PRISM^TM^ Pain Health Assessment (PHA) patient questionnaire to determine if select patient-reported behaviors can be linked by potential cause-and-effect relationships while controlling for a broad spectrum of background patient characteristics (Reed et al., manuscript in preparation). In addition, several presentations on work derived from the clinical records [16–20] have demonstrated several unique features of the data and the commitment of the team to utilize it for research.

In this report we examine some of the information content of the MPC clinical record within the context of the biopsychosocial model, and also provide an initial examination of pain outcomes. In particular, we present an exploratory factor analysis of the PHA question set to evaluate possible constructs represented by the items in the questionnaire and we provide a preliminary mapping to biological (medical), psychological, and social latent variables that would be part of a biopsychosocial phenotype model for this population. This methodology is often used to determine the factors contained within survey tools and occasionally to assess construct validity (e.g., [21, 22]). We conclude that the data available in this clinical practice is likely suitable for the determination of many pain phenotypes and outcomes. This offers the opportunity to build a base for future research and enhanced care delivery.

### Description of Electronic Health Record (EHR) and Pain Health Assessment (PHA) Tool

#### Data content of electronic health record

The types and characteristics of the data stored in the EHR at MPC are shown in Table 1. From this collection of data the patient encounter trajectory of symptom, history, finding, diagnosis, treatment, and outcome can be discerned. Once this information is extracted and analyzed, the goal will be to deduce patient phenotypes and the linkages to treatments and outcomes. All the categories denoted in Table 1 are assumed to be important for success in model creation. This report will focus on examining a portion of the PHA survey to estimate its information content and potential usefulness

**Table 1:** Estimated number of measurable variables available within the Electronic Health Records of MPC

Category	Variables	Comment
Demographic & Lifestyle	5 administrative 11 from PHA	
Treatment codes	1 to 33 per patient	Mean = 5.7
Drug codes	1 to 13 per patient	Mean = 3.4 Drugs used for procedures
Prescription Drugs	10 major classes	90 % of all Rx in top 10 classes Mean = 4.6 Rx per patient ~27 % of patients receive Rx
Diagnosis codes	1 to 17 per patient	Mean = 3.7
PHA Questionnaire	163 items representing multiple scales and sub-scales	Patient-reported status and outcomes. Additional items are included for narcotic risk, demographics and lifestyle.
Progress Notes		
Canonical sections	8	
Extractable variables	Unknown	Estimates from preliminary work would indicate at least 10 variables per section

#### Historical background: development and deployment of the PHA

As part of MPC’s initial assessment, patients are asked to complete an initial pain health assessment (iPHA). MPC utilizes the PRISM^TM^ Pain Health Assessment (PHA^TM^), developed by ProCare Systems, Inc., which is a patient self-assessment instrument that provides demographic, medical and social history, as well as patient-reported outcomes (PRO). It contains core outcome domains that evaluate the efficacy of treatments, consistent with the recommendations of the Initiative on Methods, Measurement, and Pain Assessment in Clinical Trials (IMMPACT) [23, 24]. The broad domains of the PHA are described in Table 2 with more details in Table 3. The full text of the 163 question set for the PHA are available upon request from ProCare Systems for research purposes.

**Table 2:** Categories, response types, and item counts for the iPHA and the cPHA questionnaire

PHA Categories	Response types	items	Comment
Demographic	y/n item select	5	2 items in iPHA only
Lifestyle & abuse history	Item select	11	8 items in iPHA only
Employment & disability	y/null	20	In both iPHA and cPHA
Syndromes & Diagnoses	y/null	15	In both iPHA and cPHA
Pain anatomic location	y/null	17	iPHA only
Biopsychosocial (total)	11-pt Likert	95	
iPHA only	11-pt Likert	7	Initial panel: Pain interference with daily life
cPHA only	11-pt Likert	21	Outcomes and satisfaction

iPHA: intake PHA, filled out on or near the first visit

cPHA: continuing, follow-up PHA filled out at 3 mo, 6 months, and every 6 mo thereafter

**Table 3:** PHA question numbers and descriptive label of question content

Questions	PHA (Pain Health Assessment) descriptive labels
Q1	Ethnicity
Q2	Race
Q3 A – Q	Symptoms
Q4 A – O	Syndromes and Diagnoses
Q5 A – B	Worst and Least Pain Today
Q6 A – G	Pain interfering (past 24 h) with activity, mood,walking, work, relationships, sleep, and enjoyment of life
Q7	Estimate of health
Q8 A – B	Health limiting moderate activity, strenuous activity during typical day
Q9 A – B	Health (past 4 weeks) limiting accomplishments or work
Q10 A – B	Emotional problems (past 4 weeks) limiting accomplishments or work
Q11	Pain (past 4 weeks) interfering with work
Q12 A – C	Feelings (past 4 weeks); calm and peaceful, lot of energy, downhearted and depressed
Q13	Physical or emotional health (past 4 weeks) interfering with social activities
Q14	Health compared to one year ago
Q15 A – C	Worst pain, average pain, pain right now
Q16 A – P	Activities of daily living
Q17 A	Physical activity makes me hurt more
Q17 B – D	Preconceptions; activity make me feel better, safe for me to be active, I should do normal work
Q18 A - K	Feelings (past 4 weeks). Pep, nervous, down in dumps, worn out, happy, tired, anxious, worry, angry, depressed, memory problems
Q19 A – F	Pain getting in the way of; enjoying social activities, doing social activities, family relationships, friend relationships, pleasure with family, ability to plan
Q20 A – D	Perception of control; life, handling problems, control of pain, coping with stress
Q21 A – D	Time devoted to; visiting friends, partaking in groups, enjoying hobbies, activities outside the house
Q22 A – D	Limitations (past 4 weeks), time on work or activities, accomplishments, performance
Q23	Initial pain before treatment
Q24	Average daily pain at this point in treatment
Q25	Relief received from treatments and medications
Q26 A - G	Improvement in; activity, mood, walking, work, relationships, sleep, enjoyment of life
Q27 A - K	Rating of clinical experience; time, courtesy, confidence, quality, administration, confidence of recommendations
Q28 A - M	Questions regarding employment status and disability
Q29 A - G	Specific questions regarding disability support
Q30	Marital status
Q31	Persons in household
Q32	Education
Q33	Smoking habits
Q34	Alcohol use
Q35	History of substance abuse
Q36	Family history of substance abuse
Q37	Preadolescent sexual abuse
Q38	Who completed PHA
Q39	How was PHA completed

The development of the PHA was motivated by the needs of community based physicians in the emerging field of pain medicine to demonstrate the quality and effectiveness of the care being provided. ProCare began working with the American Academy of Pain Medicine in the mid 1990’s as a beta test site for the Digimed project. Although that system failed to produce the sensitivity needed for the task, the project set the stage for the evolution of other measurement systems.

In 2004, the TOPS [25] survey was mailed to patients with a return mailing for their response. After six months of trials, the completed return rate was roughly only 30 %. In 2005, the TOPS tool was implemented in the MPC clinics. The original survey was difficult to administer without interfering with patient flow in the clinic and additionally, many scales were not sensitive enough to demonstrate changes over time.

In 2007 substantial evolutionary changes to the survey and its administration were undertaken. The goal was to develop a PRO tool that would not interfere with clinic flow or add significantly to the cost of care. The tool was composed of quality-of-life indicators, as well as additional questions assessing functional impairment, psychosocial health, and patient satisfaction. Response scales were universally formatted to an 11-point Likert scale to be sensitive enough to map incremental changes. In 2009, technology allowed for a fully functional web-based system that could be accessed from any computer or tablet device. In 2011 an assessment of narcotic risk was incorporated.

Currently, the PHA is self-administered by patients using iPads provided in the clinic at check-in or through the PRISM^TM^ portal. Information from the PHA is instantly analyzed and transferred to a Patient Summary Page dashboard for the physician and care team to inform clinical decision making in real-time during the patient visit.

#### Current deployment characteristics

Two versions of the questionnaire are used in the practice: an initial assessment version (initial PHA or iPHA) for new patients and a version for follow-up visits containing repeated measures for most iPHA questions plus the addition of specific outcome questions. (This is referred to as the cumulative PHA or cPHA.). The cPHA is also used for patients that did not complete an iPHA because their initial visit preceded its rollout. The full questionnaire covers 39 categories with a total of 163 items designed to assess pain characteristics, social and demographic characteristics, health behaviors, habits, symptoms, narcotic risk, and self-reported syndromes and symptoms. A breakdown of categories is shown in Table 2. For the initial assessment, the questionnaire is limited to 34 categories and 142 items. For the cPHA, five additional patient outcome and satisfaction categories are activated, containing 21 items, while three categories useful only for intake are deactivated, yielding a total of 36 categories and 131 items. The MPC practice typically follows patients on a long term basis, with cPHA’s administered 3 monthsnths after first treatment, 3 months later, and every 6 monthsnths thereafter.

## Methods

### Data

Michigan State University (MSU) through its Biomedical Research Informatics Core (BRIC), a division of the MSU Clinical and Translational Sciences Institute (CTSI) has entered into a Business Associates agreement with Michigan Pain Consultants (MPC) and ProCare Systems, Inc. MSU BRIC houses de-identified copies of their Pain Health Assessment questionnaire, their de-identified practice management data, and their dictated clinical progress notes on a secure workstation isolated from the internet, in compliance with HIPAA regulations and IRB requirements. The Michigan State University IRB has declared this project to have a category 4 exempt status under 45 CFR 46.101(b)(4). Questionnaires from April 1, 2010 to September, 2014 were used in this analysis because the PHA question slate remained unchanged during this span, except for the addition of the Opioid Risk Tool [26] questions in 2011. The questionnaire data obtained after this time period will be used in future work to examine test administration consistency, test-retest reliability, and stability of phenotypes and outcomes.

During the study period, approximately 30,400 patients completed one or more PHA surveys. Of this number, ~22,700 were new patients who completed the iPHA. A total of ~61,000 cPHAs were completed, which included patients who had completed the iPHA during this span and patients who had completed previous versions of the iPHA or none at all due to their much longer history with the MPC clinics. Approximately 12,600 patients had completed both an iPHA and one or more subsequent cPHAs during this span. The remainder of patients with an iPHA had no cPHA primarily because they either chose not to return or their return date was outside the study period. Furthermore, there are occasional workflow issues in the clinic that have hindered completion of cPHAs and some patients’ health prevents them from complying. Those patients with both iPHA and cPHA during this period had completed approximately 31,600 cPHAs over varying numbers of subsequent visits.

### Measures and analyses

#### Factor analysis

Preliminary exploratory factor analysis (EFA) of the PHA battery of questions (as implemented by the principle factor methodology in Stata™ v12) examined both the iPHA and cPHA repository. Cronbach’s alpha [27] and inter-item correlations were also calculated to help identify questions with similar response characteristics. After orthogonal rotation, factor assignments were made by examining eigenvalues, factor loading values, and alpha values for those observed variables with high inter-variable correlations. These assignments were then evaluated for their internal consistency and applicability as constructs within a biopsychosocial model.

#### Outcome analysis

To provide preliminary evidence for the utility of the PHA in pain outcomes, we focused on a small subset of questions. The PHA questions of interest for this analysis are:

- *Ques.1. Overall, how much relief have you received from pain treatments and medications? Please fill in the percentage that shows how much relief you have received. (0–10) 0 % - 100 %;*
- Ques.2. “*On a scale of 0–10, please rate your average pain in the past 4 weeks*”;
- Ques.3. “*Fill in the number that best describes your average daily pain at this point in treatment at the pain management center. Please rate (0–10) No pain – worst pain*”;
- Ques.4. *“On a scale of 0 to 10… Your control over your pain. (0–10) Complete – None”*

Ques.1 and Ques. 3 appear only in the cPHA. (These questions do not appear in this order or in these positions in the PHA, but have been enumerated here for easy reference.) In each of these questions, the responses are on an 11-point Likert scale ranging from 0–10. For question Ques.1, this scale was presented to patients as percent relief (0 – 100 %).

## Results

### Exploratory factor analysis

The PHA was assembled to evaluate multiple patient dimensions by deploying several question items that potentially consolidate into factors and therefore may be combined into scale scores useful as representations of constructs in models and decision support engines. While the items in the PHA were inspired by existing pain questionnaires, the reliability, validity, and construct mapping of item sets deployed in the PHA were not determined a priori in pilot studies. Rather, the decision was made by ProCare to collect large quantities of responses within the environment of a community-based. multi-site medical practice to allow the data to drive the organization of constructs from identifiable factors. Once in place, reliability and validity estimates can be made. Therefore, we begin with an unbiased examination of these items using exploratory factor analysis to identify groups of items that have high inter-item correlation, strong factor loadings, and high Cronbach alpha values. The goal is to determine which questions can be combined into relatively homogenous scales and which questions provide unique information and should stand alone. In contrast to efforts to design question batteries that can be combined into scales representing conceptual constructs (e.g., [22]), this work starts with a broad battery of questions relevant to chronic pain patients and seeks to identify subsets of questions that may represent constructs valuable in modeling phenotypes of chronic pain patients.

**Fig. 1:**
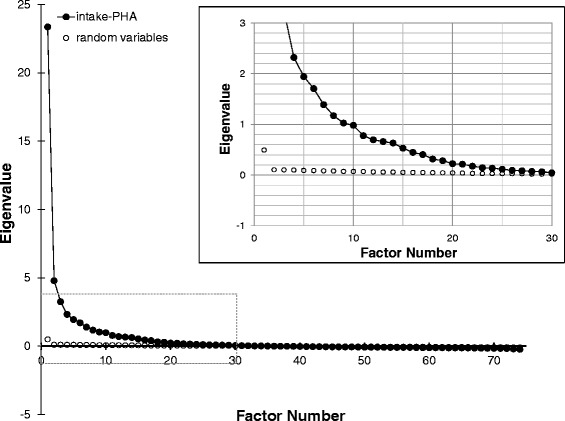
Exploratory factor analysis Scree plot. Scree plot for the exploratory factor analysis of the iPHA data set and for a data set of the same dimensions composed of random answers

As shown in Table 2, there are multiple categories in the PHA that will be important contributors to a phenotype model. As shown in Table 1, the PHA itself is only a portion of the total data repository of the EHR that will be critical to building a comprehensive model with predictive power. Within the iPHA, the biopsychosocial categories are composed of 74 questions that are asked before treatment. Examining the suitability of factor analysis for this set using the KMO test yielded a value of 0.96, indicating that 96 % of the variability in the data may be caused by underlying factors. Therefore, EFA was performed and yielded eigenvalues with a pattern in the scree plot that suggested as many as 30 factors may be significant using the threshold criterion of Cattell (1966) [28]. This number was deemed overly large and a more conservative approach was taken. Using the random matrix criterion of Horn (1965) [29], a full set of random answers (ranging over 0 – 11) were constructed for all 74 variables and all patients. Factor analysis of this data set yielded eigenvalues plotted in Fig. 1, together with the eigenvalues for the iPHA factor analysis. If we assume that all factors worth considering should lie above the highest eigenvalue of the random set, then 14 or 15 factors are likely to be important (see inset of Fig. 1).

Choosing 15 factors for the analysis yielded 15 identifiable clusters with two or more questions per factor (Table 4). Seven of the 74 questions did not fall strongly into any factors (loadings < 0.4) and were uncorrelated with each other, suggesting that they offer unique, independent information. These are shown in Table 5.

**Table 4:** Results of exploratory factor analysis on ~ 22,700 iPHA questionnaire responses

Factor	Informal factor description	# of items	Mean loading	Mean alpha	Mean communality	Variables in factor
1	Pain on ADL - torso	9	0.726	0.945	0.726	Q6-C, Q8-B, Q16-I, Q16-J, Q16-K, Q16-L, Q16-M, Q16-N, Q16-O
2	Pain affecting work	8	0.600	0.943	0.758	Q8-A, Q9-A, Q9-B, Q11, Q22-A, Q22-B, Q22-C, Q22-D
3	Mental health	8	0.565	0.901	0.563	Q12-C, Q18-B, Q18-days, Q18-G, Q18-H, Q18-I, Q18-J, Q18-K
4	Pain on ADL – fine motor	6	0.644	0.894	0.618	Q16-A, Q16-B, Q16-C, Q16-D, Q16-E, Q16-H
5	Pain intensity	6	0.670	0.865	0.554	Q5-A, Q5-B, Q6-F, Q15-A, Q15-B, Q15-C
6	Social interactions	4	0.554	0.848	0.621	Q19-C, Q19-D, Q19-E, Q19-F
7	Coping ability	3	0.692	0.900	0.745	Q20-A, Q20-B, Q20-D
8	Effects of pain – past 24 h	5	0.532	0.900	0.686	Q6-A, Q6-B, Q6-D, Q6-E, Q6-G
9	Social activities	4	0.534	0.811	0.530	Q21-A, Q21-B, Q21-C, Q21-D
10	Mental state affecting work	2	0.791	0.964	0.878	Q10-A, Q10-B
11	Pain affecting hobbies	2	0.452	0.930	0.816	Q19-A, Q19-B
12	Vitality - pep	3	0.751	0.738	0.485	Q12-A, Q12-B, Q18-A
13	Pain affecting jaw	2	0.666	0.864	0.672	Q16-F, Q16-G
14	Vitality - tiredness	2	0.632	0.825	0.520	Q18-D, Q18-F
15	Pain-work attitude	3	0.446	0.690	0.463	Q17-B, Q17-C, Q17-D

**Table 5:** Questions with no significant loadings on any of the 15 factors

Question	Paraphrased question content
Q7	In general would you say your health is: (0–10), (excellent – poor)
Q13	In past 4 weeks how much has health or emotions limited social activity (0–10), (none of time – all of time)
Q14	Compared to one year ago, how do you rate your health (0–10), (much better – much worse)
Q16-P	Does your health now limit you in sitting: (0–10), (not at all – a lot)
Q17-A	Physical activity makes me hurt more (0–10), (completely agree – completely disagree)
Q18-E	How are you feeling in past 4 weeks: Have you been a happy person? (0–10), (none of time – all of time)
Q20-C	Your control over your pain (0–10), (complete – none)

In Table 4, a summary of the factor metrics and informal descriptions are given for iPHA exploratory factor analysis. In the analysis of the iPHA, the subject to item ratio is approximately 300:1. This is the very high end of typical studies and well beyond the recommended 20:1 ratio [30], therefore the results should be robust. Some of the findings among the factors are that there was a separation between performing and enjoying activities; coping was separate from other mental health characteristics; and, coarse motor activities were separate from fine motor activities. Work and hobbies separated from each other and pain and pain effects separated. The high mean loadings and Cronbach alphas support this pattern of item consolidation. As an informal examination of test-retest validity, EFA of the cPHA questions that are in common with the iPHA questions yielded almost identical factoring with only two questions changing factor assignments (not shown). This was due to relatively weak loading values (between 0.4 and 0.5) in each of these two cases, in both the iPHA and cPHA analyses.

The communality values in Table 4 are indicators of the average fraction of the variance that the factors contribute to the question responses. It is clear from these values that while the common factor loadings account for substantial variance, unexplained variance remains. This is attributable to unique factors – components specific to each question plus experimental error. These unique factors are by definition uncorrelated with each other and their non-error components contribute to the information content of each question. This information may be important to any model of patient phenotypes and must be included in model construction. Techniques such as structural equation modeling (SEM) account for this unique information and will be employed in future work during phenotype model design and testing.

**Fig. 2:**
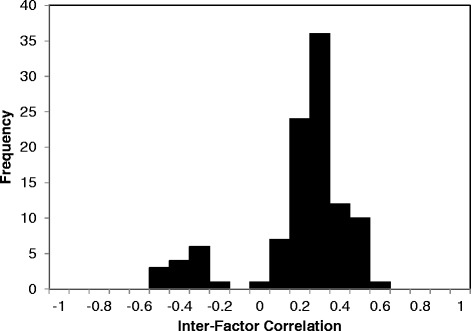
Frequency distribution of the correlation matrix. Frequency distribution of the correlation matrix values for the scales derived from the 15 factors shown in Table 3. All possible combinations of the scales generate 105 correlation values

To examine the degree of independence among the 15 identified factors from the iPHA analysis (Table 3), scales were created for each factor by computing the mean of each result variable within each factor for each patient, creating 15 new variables per patient. A frequency distribution of the correlations between all possible combinations of the 15 factor variables is shown in Fig. 2. Most of these correlations (~92 %) are between -0.5 and 0.5, indicating weak to moderate correlations and high independence among factors, but there are a few values (7.6 %) above 0.5 indicating that future modeling may need to include correlation terms between a few factors. On the other hand, there are no notably high correlations between factor variables suggesting that the identified factors can be utilized as initial estimates for measures of patient characteristics.

In the follow-up questionnaire, the 21 questions unique to the cPHA (Q23 – Q27) formed two factors plus three questions that did not have strong loadings. Two of those outcome questions (Q24, Q25) are analyzed below (Ques.1 and Ques.3). With regard to the two identified factors, one is composed of 11 questions related to patient satisfaction with the clinical experience (Q27). The other contains seven questions regarding biopsychosocial health improvements after treatments (Q26). These question batteries have alpha values of 0.96 and 0.94, respectively, and in a cPHA factor analysis they consolidate into factors with mean loadings of 0.85 and 0.78, respectively. In order to fully examine the factors for the cPHA, the cases must be sorted into groups denoted by which cPHA in a patient’s treatment it is. Currently, patients range from having only one cPHA on file (20,300) to ten on file (42). Full factor analyses for these groups are outside the scope of this report.

### Outcomes analysis

In the previous section, a portion of the PHA was shown to contain identifiable structure that would help populate components of a person-in-pain phenotype model. To be a comprehensive dataset, it must also contain viable outcomes information to allow the full phenotype-treatment-outcome model to be constructed. A sample of outcome measures is explored in this section.

**Fig. 3:**
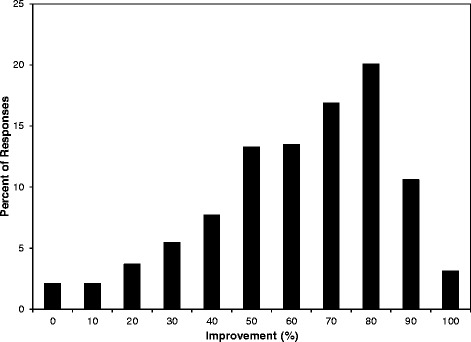
Responses to Question 1. Responses to Ques.1 from 61,161 patients who have completed the cPHA

**Fig. 4:**
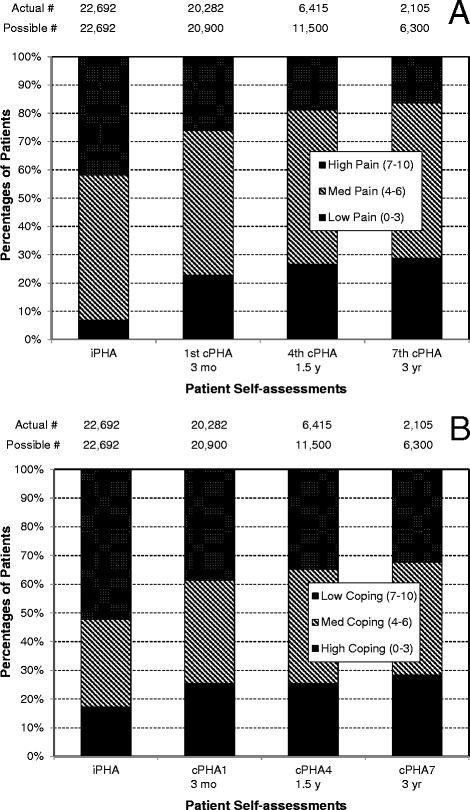
Responses to Questions 2–4. Responses to questions Ques.2, Ques.3, and Ques.4 for intake and continuing PHA patient surveys. **a** The 11-point pain scale was consolidated into three ranges (low pain: 0 to 3; medium pain: 4 to 6; and high pain: 7–10). Ques.2 was used for the intake PHA, while the outcome Ques.3 was used for all the cPHA. The number of patients that have completed the respective surveys are shown at the top of the figure as “Actual #”. The possible number who could have completed 1 or more cPHAs is shown at the top of the figure as “Possible #”. The nominal times between the iPHA and subsequent cPHAs are shown below each column. **b** The 11-point coping scale for Ques.4 was consolidated into three ranges (high coping: 0 to 3; medium coping: 4 to 6; and low coping: 7–10). The number of patients that have completed the respective surveys are shown at the top of the figure as “Actual #”. The possible number who could have completed one or more cPHAs is shown at the top of the figure as “Possible #”. The nominal times between the iPHA and subsequent cPHAs are shown below each column

Responses to question Ques.1 (see Methods section) strongly indicated that patients experienced benefit from the care received. This is shown in Fig. 3 as the frequency distribution for responses to this question. This effect is corroborated by responses to questions Ques.2, Ques.3, and Ques.4. In Fig. 4a it is shown that the fraction of patients with lower overall pain levels increased with time as documented in their subsequent cPHA responses. In Fig. 4b, a similar effect is seen for the patient’s ability to cope with pain. The trend was demonstrated by consolidating the 11-point pain scale into three broad ranges (low pain: 0 to 3; medium pain: 4 to 6; and high pain: 7–10) for Ques.2 and Ques.3, and (high coping: 0 to 3; medium coping: 4 to 6; and low coping: 7–10) for Ques.4. With increasing visits, there is a consistent reduction in the percentage of with high pain levels and low coping levels. These are aggregate results and the numbers of patients with 1, 4 and 7 cPHAs on file are shown at the top of the chart and labeled as “Actual #”. Due to the continual influx of new patients over the four years of the stable version of the PHA, not all patients have been in the practice long enough to have reached their 4th or 7th cPHA. The maximal possible numbers who could have reached their 4th or 7th cPHA are shown above the charts as “Possible #”. The approximate timings of the PHAs are shown along the bottom of the figure. These descriptive results are not meant to be conclusive but are presented only to demonstrate the breadth of the data and its capacity to support the goal of phenotype construction and/or hypothesis testing.

While aggregate results are enlightening, longitudinal intra-patient responses offer a more robust examination of treatment responses. Therefore, within-patient pain outcomes were examined in the patient population that had both iPHA and cPHA responses on file from the April 2010 to September 2014 time period. This was approximately 12,600 patients. From this group two subpopulations were identified: one that had initial responses to Ques.2 within the range 1–9 (approximately 11,900 patients); and one that had initial responses only in the range 7–9 (approximately 4600 patients). Question #2 requests a patient’s pain level within the previous four weeks. We used the responses to this question in the iPHA as an indicator of starting pain levels. Outcomes from treatment were examined by calculating the numeric change in responses to Ques.2 in the subsequent visits when the cPHA was filled out, and by examining the responses to Ques.3, which is specifically worded as an outcome question and exists only in the cPHA.

**Fig. 5:**
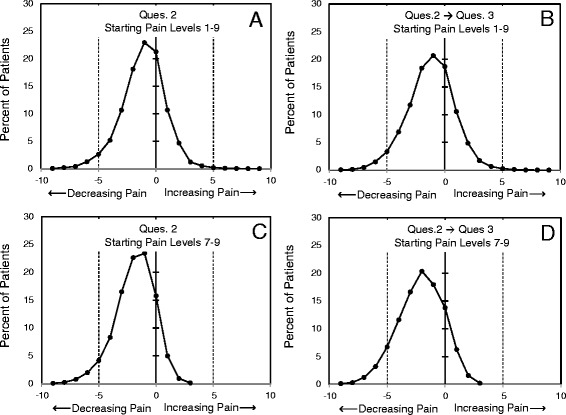
Intra-patient changes in responses to questions of pain levels. Intra-patient changes in responses to questions of pain levels. **a** Patients with starting pain levels (Ques.2) in the range 1–9 are evaluated for mean pain level change as determined by answers to Ques.2 in the cPHA surveys. **b** Patients with starting pain levels (Ques.2) in the range 1–9 are evaluated for mean pain level change as determined by answers to Ques.3 in the cPHA surveys. **c** Patients with starting pain levels (Ques.2) in the range 7–9 are evaluated for mean pain level change as determined by answers to Ques.2 in the cPHA surveys. **d** Patients with starting pain levels (Ques.2) in the range 7–9 are evaluated for mean pain level change as determined by answers to Ques.3 in the cPHA surveys. Characteristics of the distributions are given in Table 4

To obtain an appreciation for the range of responses rather than simple averages, the distributions of patient outcomes were calculated. The distributions of the changes in patient pain levels with time are shown in Fig. 5. For display simplicity, values are plotted as if continuous. In panel A, the distribution of the differences between initial responses to Ques.2 and subsequent responses to Ques.2 are shown for all patients with starting pain within the range of 1–9. The pain difference was calculated by averaging all the cPHA responses for a particular patient before subtracting the initial pain level. This approach is repeated in panels B, C, and D of Fig. 5. In panel b, the outcome question Ques.3 of the cPHA is used as a comparison to the initial pain responses of Ques.2 in the iPHA. As in panel A, these are calculated for patients with initial pain levels in the range of 1–9. Panels C and D repeat the approach used in panels A and B, except the calculations are limited to high-pain patients; those with initial pain levels in the range of 7-9. Patients with initial pain levels of zero and ten are excluded because scale ceiling and floor values can only allow changes in one direction and this has the potential to skew the results. Excluding the patients with the extreme values of 0 and 10 represented only approximately 3 % patients with both iPHA and cPHA surveys in their clinical record.

The distributions within Fig. 5 are all fairly smooth, unimodal, and significantly shifted from zero (p < 0.001), indicating substantial improvements in pain levels. Metrics for these distributions are given in Table 6. While the best outcomes are seen for those patients with the highest starting pain, a high percentage of patients experienced a reduction in pain. This is shown as the percentage of “Patients Improving” in Table 6. The distributions of Fig. 5 also reveal that a substantial number of patients reported reductions in pain levels of 3, 4, 5, and even 6 pain units. It should be noted that the larger effects in the high pain patients must be considered preliminary because there will be some tendency for responses to migrate to the center of the Likert scale unrelated to treatment. A formal evaluation of the size of this effect is outside the scope of this current presentation.

**Table 6:** Characteristics for distributions in Fig. 5

	Mean^a^	Std Dev	Std Error^b^	Patients Improving^c^	N
Starting Pain 1-9					
Panel A	−1.10	1.89	0.017	71.98 %	11,927
Panel B	−1.21	2.01	0.018	72.58 %	11,927
Starting Pain 7-9					
Panel C	−1.85	1.72	0.030	88.17 %	4598
Panel D	−2.10	1.95	0.029	85.87 %	4598

^a^Values are in units of the 11-point pain scale. Negative values indicate a shift to less pain

^b^Z-scores calculated from the Means and Standard Errors indicate that these distributions are significantly different from identical distributions centered at zero (p-values < <0.001)

^c^“Patients Improving” is calculated from a normal distribution using the means and standard deviations of the distributions of Fig. 4. The area to left of zero is taken as representing patients improving, provided as a percentage, under the assumption of a continuous scale

## Discussion

Health records from a community-based pain medicine practice have been described and a preliminary evaluation of patient-reported status and outcomes has been performed for patients seen between April, 2010 and September, 2014. The patient population is constrained by the demographics, socio-economic profile, and local referral patterns of the region. In this case, the 7 community clinics of Michigan Pain Consultants represent an upper Midwest USA region serving 6 counties in West Michigan containing medium-sized urban, suburban and rural environments. The patients seen by this practice are those with commercial or government medical insurance or have the means for private pay. As such, the findings, while broad, cannot be generalized to large urban centers or to the poor without further work to show that the resulting phenotypes cross these boundaries.

This work is part of a larger study designed to construct a phenotype – treatment – outcome model for pain medicine. Our working hypothesis is that knowing phenotype profiles for pain patients will help predict appropriate treatments associated with better outcomes and that this type of data can be extracted from community clinical practice, where most chronic pain is treated. A university-community partnership was established to provide a foundation for this study. The first goal was to determine if the pain clinic medical record database (EHR) contained sufficient information to construct a model. The MPC EHR is composed of practice management data, patient questionnaires, and physician progress notes with a longitudinal component available for many patients.

In this study, we show that the biopsychosocial component of the patient questionnaire contains identifiable factors that will allow combining question items into measures of interest for a phenotype model. It should be noted that no inferential statistical methods are available for exploratory factor analysis, thus there is a subjective element to the choice of the number of eigenvalues and acceptance/rejection criteria for correlations, loadings, and Cronbach alpha values. Nevertheless, from exploratory factor analysis, preliminary models can be constructed and confirmatory factor analysis can provide the statistical tests to evaluate these models. We will use the data acquired after September, 2014 for this type of work.

It will also be important to identify the factors (latent variables) in the practice management data, the progress notes, and in the remainder of the PHA data. Larger models can then be constructed, initially tested with CFA and then comprehensively tested with structural equation modeling (SEM) [31]. It is desirable that the larger models encompass the full patient encounter axis of symptom, history, finding, diagnosis, treatment, and outcome. Once the latent factors are identified, the next step toward phenotype definitions will be determining the latent classes that the patients cluster into. Whereas factor analysis groups items into common factors, latent cluster (class) analysis identifies groups of patients having similar patterns of responses to the items (or similar patterns of factor scores) using one of several available methods (e.g., [32, 33]). These clusters will be our initial phenotypes. Preliminary examinations of patient responses to a few subsets of factors indicate that strongly orthogonal clusters of patients exist in the iPHA set, using the k-means clustering algorithm. A full analysis of clustering will be the subject of a follow-up paper. After the phenotype models are derived and validated. they can be used to generate hypotheses for future research, which can provide the basis for evidence-based medicine and decision support tools.

The preliminary examination of pain outcomes showed that pain indicators can be followed with time. In the analysis of the patient outcomes it is useful to note that the mean improvement of pain levels across repeated visits was approximately one to two units on an eleven-point scale. This degree of improvement was consistent with previous validation studies on small patient numbers utilizing other pain scale tools [34–36]. This suggests that both the PHA responses and the population being treated are typical of the chronic pain environment. Furthermore, it was shown that patient improvement increased with visit number and patients with the highest initial pain levels reported the greatest improvement. This indicates that the patients are improving and therefore the practice and its data reservoir should meet the needs of our long-term study goals. In addition to the specific outcome questions examined in this study, other outcome metrics can be derived from the longitudinal trajectory of patient responses to all questions in the cPHA. This can supply supportive evidence for changes in well-being and quality of life that are part of the total picture of pain management.

This approach is important for the field of pain medicine and for the patients receiving therapy. The use of systems like the PRISM^TM^ care management clinical outcomes tool helps to assess the efficacy of interventional treatments and helps guide physicians in the use of opiates and other medications^1^. There are many concerns with opioid therapies and how they are monitored in chronic pain patients [37]. For many patients they are an important part of managing their pain . For others, there are abuse problems. Similarly, interventional therapies help many patients but not all [38]. Documenting the biopsychosocial, patient and physician perspectives enhances decision-making which can lead to more personalized care that can address these differences. ProCare has already developed tools that have been utilized to determine the best use of opioids and to garner outcomes from procedures applied to populations of patients. This information has been fed back to the physicians resulting in a reduction of imaging and narcotic usage while maintaining patient satisfaction, thus leading to increased value and safety. This has helped establish better working relationships with payers and is a potent tool for patient advocacy.

Finally, it must also be noted that many patients stop returning after varying numbers of visits. This leads to many patients with only the iPHA on file and/or only a few cPHAs on file. The reasons for patients not returning are probably many. After an initially scheduled follow-up visit, a patient may not return because their pain is adequately under control, or they did not receive any relief, or they left the area, changed insurance plans or have other financial barriers, or they are deceased. It is important that we determine the size and characteristics of these sub-populations to make meaningful longitudinal predictions of outcomes. These patient decisions will also need to be part of the chronic pain phenotype profile. This will be one focus of future work based on sampling surveys.

## Conclusions

In conclusion, early results are presented that lay the groundwork for extracting patient and physician perspectives in a community pain medicine specialist practice. We have demonstrated that multiple biopsychosocial variables and pain level outcomes are being captured by the various tools used by the practice.. We believe that these data can be used to begin building phenotypes for persons in chronic pain and the linkages to treatments and outcomes that, in turn, can be used to facilitate evidence-based, personalized treatment of pain in community settings.

## Abbreviations

MPC: Michigan pain consultants
MSU: Michigan State University
CTSI: Clinical and translational sciences institute
BRIC: Biomedical research informatics core
SOAP note: Subjective objective assessment plan progress note format
PHA: Pain health assessment
iPHA: Pain health assessment – intake form
cPHA: Pain health assessment – continuing or follow-up evaluation form
IMMPACT: Initiative on methods, measurement, and pain assessment in clinical trials
SF-36: 36-item short-form health survey in medical outcomes study
TOPS: Treatment outcomes in pain survey
EHR: Electronic health record
PRO: Patient-reported outcomes
EFA: Exploratory factor analysis
CFA: Confirmatory factor analysis
SEM: Structural equation modeling
Ques.1: Ques.2, Ques.3, Ques.4, four questions from the PHA

## Footnotes

1 ProCare Systems has been in substantive discussions with the State of Michigan Department of Community Health to utilize the PRISM System and PHA to form the foundation of a care management system to assist physicians prescribing opioids for chronic pain patients. This system will also be utilized to help better manage the use of Suboxone therapy for treatment of addiction disorders.

## References

[1] Institute of Medicine: *Relieving Pain in America A Blueprint for Transforming Prevention, Care, Education and Research*. National Academies Press, Washington, D.C. 2011. 22553896

[2] Gaskin DJ, Richard P (2012). The economic costs of pain in the United States. J Pain.

[3] Kennedy J, Roll JM, Schraudner T, Murphy S, McPherson S (2014). Prevalence of persistent pain in the U.S. adult population: new data from the 2010 national health interview survey. J Pain.

[4] Manchikanti L (2008). Evidence-based medicine, systematic reviews, and guidelines in interventional pain management, part I: introduction and general considerations. Pain Physician.

[5] Patsopoulos N (2011). A pragmatic view on pragmatic trials. Dialogues Clin Neurosci.

[6] Friedly JL, Bresnahan BW, Comstock B, Turner JA, Deyo RA, Sullivan SD (2012). Study protocol- Lumbar Epidural steroid injections for Spinal Stenosis (LESS): a double-blind randomized controlled trial of epidural steroid injections for lumbar spinal stenosis among older adults. BMC Musculoskelet Disord.

[7] Weng C, Li Y, Ryan P (2014). A distribution-based method for assessing the differences between clinical trial target populations and patient populations in electronic health records. Appl Clin Inform.

[8] Schiller JS, Lucas JW, Peregoy JA: Summary health statistics for U.S. adults: National Health Interview Survey (2011). National center for health statistics. Vital Health Stat.

[9] Sarkar IN (2010). Biomedical informatics and translational medicine. J Transl Med.

[10] Giordano J, Abramson K, Boswell MV (2010). Pain assessment: subjectivity, objectivity, and the use of neurotechnology. Pain Physician.

[11] Manchikanti L, Singh V, Datta S, Cohen SP, Hirsch JA (2009). Comprehensive review of epidemiology, scope, and impact of spinal pain. Pain Physician.

[12] Witkin LR, Farrar JT, Ashburn MA (2013). Can assessing chronic pain outcomes data improve outcomes?. Pain Med.

[13] Gatchel RJ, Peng YB, Peters ML, Fuchs PN, Turk DC (2007). The biopsychosocial approach to chronic pain: scientific advances and future directions. Psych Bull.

[14] Ablin JN, Buskila D (2013). Personalized treatment of pain. Curr Rheumatol Rep.

[15] Juckett DA (2012). Method for determining the number of documents needed for a gold standard corpus. J Biomed Inform.

[16] Davis FN, Mossey JM, Gostine ML, Risko R, Cubbage C: Clinical Assessment of Physical and Psychosocial Pathology in Pain Medicine. *Pain Med* 2012, 13(2): Annual Meeting Abstracts 319.

[17] Thompson PA, Davis FN, Reed PL, Mossey JM, Gostine ML, Risko R: Effect of Pain on Work, Daily Life Tasks, and Activities Among Patients Seeking Treatment for Chronic Pain. *Pain Med* 2012, 13(2): Annual Meeting Abstracts, 330.

[18] Davis FN, Risko, R, Lubinsky GT, Cubbage C, Gostine ML: Psychosocial, Functional, and Quality of Life Status Associated with Opioid Risk. *Pain Med* 2013, 14(4): Annual Meeting Abstracts, 571.

[19] Gostine ML, Davis FN, Gostine A, Lubinsky GT, Gostine D, Risko R: Correlation of Misuse of Narcotics with Opioid Risk Tool and Pain Health Assessment: An Analysis of 16,000 Patients. *Pain Med* 2013, 14(4): Annual Meeting Abstracts, 573.

[20] Gostine ML, Davis FN, Risko, R: Risk assessment in the digital age: Developing meaningful screening tools for opioid prescribers. *Practical Pain Management*, 14: 56–61.

[21] Sipps GJ, Howard LC (2013). Exploratory factor analysis of the pain outcomes profile. J Clin Psychol Med Settings.

[22] Jacobs KS, Roodenburg J (2014). The development and validation of the self-report measure of cognitive abilities: a multitrait-multimethod study. Intelligence.

[23] Turk DC, Dworkin RH, Allen RR, Bellamy N, Brandenburg N, Carr DB (2003). Core outcome domains for chronic pain clinical trials: IMMPACT recommendations. Pain.

[24] Dworkin RH, Turk DC, Farrar JT, Haythornthwaite JA, Jensen MP, Katz NP (2005). Core outcome measures for chronic pain clinical trials: IMMPACT recommendations. Pain.

[25] Rogers WH, Wittink HM, Ashburn MA, Cynn D, Carr DB (2000). Using the “TOPS”, an outcomes instrument for multidisciplinary outpatient pain treatment. Pain Med.

[26] Webster LR, Webster RM (2005). Predicting aberrant behaviors in opioid-treated patients: preliminary validation of the opioid risk tool. Pain Med.

[27] Cronbach L (1951). Coefficient alpha and the internal structure of tests. Psychometrika.

[28] Cattell RB (1966). The scree test for the number of factors. Multivar Behav Res.

[29] Horn J (1965). A rationale and test for the number of factors in factor analysis. Psychometrika.

[30] Costello A, Osborne J (2005). Best practices in exploratory factor analysis: four recommendations for getting the most from your analysis. Pract Assess Res.

[31] Beran TN, Violato C (2010). Structural equation modeling in medical research: a primer. BMC Research Notes.

[32] Mun EY, von Eye A, Bates ME, Vaschillo EG (2008). Finding groups using model-based cluster analysis: heterogeneous emotional self-regulatory processes and heavy alcohol use risk. Dev Psych.

[33] Skinner HA, Blashfield RK (1982). Increasing the impact of cluster analysis research: the case of psychiatric classification. J Consult Clin Psych.

[34] Jensen MP, Turner JA, Romano JM, Fisher LD (1999). Comparative reliability and validity of chronic pain intensity measures. Pain.

[35] Tan G, Jensen MP, Thornby JI, Shanti BF (2004). Validation of the brief pain inventory for chronic nonmalignant pain. J Pain.

[36] Turner JA, Shortreed SM, Saunders KW, Leresche L, Berlin JA, Von Korff M (2013). Optimizing prediction of back pain outcomes. Pain.

[37] Manchikanti L, Atluri S, Trescot AM, Giordano J (2008). Monitoring opioid adherence in chronic pain patients: tools, techniques, and utility. Pain Physician.

[38] Manchikanti L, Cash KA, McManus CD, Pampati V, Fellows B (2012). Results of 2-year follow-up of a randomized, double-blind, controlled trial of fluoroscopic caudal epidural injections in central spinal stenosis. Pain Physician.

